# The salivary microbiome is altered in the presence of a high salivary glucose concentration

**DOI:** 10.1371/journal.pone.0170437

**Published:** 2017-03-01

**Authors:** J. Max Goodson, Mor-Li Hartman, Ping Shi, Hatice Hasturk, Tina Yaskell, Jorel Vargas, Xiaoqing Song, Maryann Cugini, Roula Barake, Osama Alsmadi, Sabiha Al-Mutawa, Jitendra Ariga, Pramod Soparkar, Jawad Behbehani, Kazem Behbehani

**Affiliations:** 1 Department of Applied Oral Sciences, the Forsyth Research Institute, Cambridge, Massachusetts, United States of America; 2 The Dasman Diabetes Institute, Kuwait City, Kuwait; 3 Ministry of Health, Kuwait City, Kuwait; 4 Kuwait University, Faculty of Dentistry, Kuwait City, Kuwait; University of Florida, UNITED STATES

## Abstract

**Background:**

Type II diabetes (T2D) has been associated with changes in oral bacterial diversity and frequency. It is not known whether these changes are part of the etiology of T2D, or one of its effects.

**Methods:**

We measured the glucose concentration, bacterial counts, and relative frequencies of 42 bacterial species in whole saliva samples from 8,173 Kuwaiti adolescents (mean age 10.00 ± 0.67 years) using DNA probe analysis. In addition, clinical data related to obesity, dental caries, and gingivitis were collected. Data were compared between adolescents with high salivary glucose (HSG; glucose concentration ≥ 1.0 mg/d, n = 175) and those with low salivary glucose (LSG, glucose concentration < 0.1 mg/dL n = 2,537).

**Results:**

HSG was associated with dental caries and gingivitis in the study population. The overall salivary bacterial load in saliva decreased with increasing salivary glucose concentration. Under HSG conditions, the bacterial count for 35 (83%) of 42 species was significantly reduced, and relative bacterial frequencies in 27 species (64%) were altered, as compared with LSG conditions. These alterations were stronger predictors of high salivary glucose than measures of oral disease, obesity, sleep or fitness.

**Conclusions:**

HSG was associated with a reduction in overall bacterial load and alterations to many relative bacterial frequencies in saliva when compared with LSG in samples from adolescents. We propose that hyperglycemia due to obesity and/or T2D results in HSG and subsequent acidification of the oral environment, leading to a generalized perturbation in the oral microbiome. This suggests a basis for the observation that hyperglycemia is associated with an increased risk of dental erosion, dental caries, and gingivitis. We conclude that HSG in adolescents may be predicted from salivary microbial diversity or frequency, and that the changes in the oral microbial composition seen in adolescents with developing metabolic disease may the consequence of hyperglycemia.

## Introduction

Potentially outnumbering human cells 10:1, the millions of bacteria, archaea, microbial eukaryotes, and viruses that inhabit the human body, collectively known as the microbiome, display a vast biological and functional diversity. Over the past 10 years, it has become clear that defining a healthy human microbial state at various sites (e.g., oral cavity, intestines, skin) is a critical step for discovering how variations in the microbiome may contribute to or cause a wide range of human diseases [1]. Indeed, it is still unclear whether the differences in the human microbiome that are seen in many disease states are a symptom of the disease or part of the underlying etiology [2]. The Human Microbiome Project, and other related research efforts in both industry and academia (including our own [3–5]), are now striving to understand and clarify the variations in microbial communities found in people in conditions of both health and disease [6].

The oral microbiome is one of the most diverse microbial communities in the body, with as many as 700 species of bacteria colonizing the hard surfaces of teeth and the soft tissues of the oral mucosa [7, 8]. Although there are substantial differences in the bacterial diversity of the oral microbiome between healthy people, the bacterial diversity of the oral microbiome remains relatively constant for any given person over time when that person remains in a state of health [9–11]. As with other body sites in which the human microbiome has been studied, long-term changes in bacterial diversity, frequency, and count in the oral cavity are observed in a number of chronic disease states. Any condition resulting in xerostomia [12], such as Sjogren’s disease and radiation therapy to the head [13], has the potential to alter salivary bacterial parameters. Other conditions such as kidney disease [14], some cancers [4, 15], cardiovascular diseases [16], and obesity [3, 17] have also been associated with changes in salivary bacterial parameters. Interestingly, type II diabetes mellitus (T2D) has been associated with clear changes in bacterial diversity and frequency in supragingival plaque in a few studies; however, an association between T2D and changes in salivary bacterial parameters is less clear [18, 19].

The prevalence of metabolic disease and T2D in adolescents appears to be increasing. The prevalence of T2D among U.S. children and adolescents in 2009 was 0.46/1000 [20], a 30% increase since 2001. Hyperglycemia is pathognomonic for diabetes. Values for fasting blood glucose greater than 100 mg/dL but less than 124 mg/dL are considered evidence of the prediabetic condition known as impaired fasting glucose [21]. Sustained values of fasting blood glucose greater than 125 mg/dL are considered diagnostic of diabetes. Such high levels of blood glucose are associated with high levels of salivary glucose, which has been promoted as a useful salivary biomarker for T2D [22]. To examine potential changes in the count and/or diversity of the bacterial species present in saliva in adolescents with high and normal concentrations of salivary glucose, we collected and analyzed whole saliva samples from 8,173 10-year old Kuwaiti adolescents. This population is of particular interest because Kuwaiti adults have one of the highest levels of T2D in the world [23], making this a high-risk adolescent population for T2D. Our previous work has indicated that, in this population, fasting salivary glucose concentrations of less than 1 mg/dL correlate with plasma concentrations of less than 100 mg/dL (normal, healthy range). Fasting salivary glucose concentrations above 1 mg/dL indicate plasma glucose concentrations of 100 mg/dL or higher (hyperglycemic) [24]. Therefore, we used the whole genomic DNA probe method commonly referred to as the “checkerboard assay” to analyze the salivary microbiota of these Kuwaiti adolescents, comparing differences in overall bacterial load, bacterial species counts, and the relative frequency of bacterial species between the group of samples with normal salivary glucose concentration and those with high salivary glucose concentration.

## Materials and methods

### Study population

The Kuwait study population and design have been previously described [25]. All of the participants enrolled were native Kuwaitis in 4th or 5th grade. A total of 8,317 adolescents participated in the study during 182 visits to 138 Kuwaiti schools made by study personnel between October 2, 2011 and May 15, 2012. The focus of this analysis is saliva samples from 8,173 adolescents in which salivary glucose concentration, bacterial counts, and relative bacterial frequencies were all measured. The study was approved by the Dasman Diabetes Institute Ethical Review Committee in Kuwait. Arabic language written informed consent was signed by parents/guardians and participant assent was signed on the day of their evaluation.

### Clinical examination

All clinical data were captured on tablet computers (iPad^®^, Apple Corporation, Cupertino, CA, USA). Height in centimeters was measured using a stadiometer, and weight in kilograms was measured using a digital scale. Waist circumference was measured at the midpoint between the bottom of the rib cage and above the top of the iliac crest. Body Max Index (BMI) was calculated by dividing body weight in kilograms by height in meters squared. Body weight categories were determined from BMI percentile using International Diabetes Federation values [26] as follows: underweight, <5th percentile; normal weight, 5th–84th percentile; overweight, 85th–94th percentile; obese >95th percentile. Systolic and diastolic blood pressure were measured using pediatric blood pressure cuffs. High blood pressure was defined as systolic ≥130 or diastolic ≥ 85 mmHg [26]. Fitness was measured by heart rate elevation following a standardized exercise [27]. Weekday sleep was self-reported.

Oral examinations were conducted by dentists assisted by trained nurses using portable dental chairs, halogen lights and intraoral mirrors [28]. No radiographic images were taken for this study, and no dental explorers were used. For each participant, the examiner recorded the number of primary teeth, the number of permanent teeth, the number of teeth with fillings, and the number of teeth with visible unfilled decay.

### Saliva collection

Saliva was collected from all participants between 8:30 am and 9:30 am under fasting conditions [25]. Each participant rinsed with and swallowed 15 mL of water before saliva collection. Adolescents were given a dated, labeled, sterile, 15-mL plastic screw-top centrifuge tube (Product #430791, Corning Incorporated Life Sciences, Tewksbury, MA, USA). Whole saliva (approximately 3 mL) was collected while keeping the tube on ice by having the participant drool into the screw-top tube. A staff monitor recorded the start time of the saliva collection, verified that approximately 3 mL was collected from each participant, recorded the stop time for each participant, and transferred the labeled tube to an ice bath for temporary storage. The salivary flow rate was computed by dividing the tarred weight of the saliva collection tube by the difference in the start and stop collection times.

### Microbial assay

Salivary microbiota were assayed using the whole genomic DNA probe method commonly referred to as the “checkerboard assay” [5]. By this method, numbers of bacteria are determined by comparison with linear regression of response from 10^5^ and 10^6^ standards for each probe. Assays were conducted using a 0.2 mL whole saliva sample obtained before centrifugation from each subject. Cell wall disruption was performed by boiling after adding 0.1 mL of 0.5 N NaOH and neutralizing by addition of 5 M ammonium acetate. It should be noted that dead cells with intact DNA can be measured by this technique but values obtained have acceptable association with those made by culture [29]. Samples were applied to the surface of a nylon membrane in a Minislot^™^ device (Immunetics, Cambridge, MA, USA) and evaluated by DNA probes to 42 species. Bacterial DNA were fixed to the membrane by ultraviolet exposure. Bacterial numbers were determined by image analysis of scanned samples (Typhoon^™^ Molecular Imager, GE Healthcare Life Sciences, Pittsburgh, PA, USA) using a covalently-bound fluorescent marker (AttoPhos^®^, Amersham Life Sciences, Arlington Heights, IL, USA). On each membrane, a mixture of DNA from each probe species at concentrations equivalent to 10^5^ and 10^6^ cells was applied to provide quantitation standards for each probe species. The concentrations of DNA probes used was adjusted to detect approximately 10^4^ bacteria (sensitivity) with 93.5% of cross-reactions exhibiting less than 5% of the homologous probe signal (specificity) [5]. The genus, species, and source of the bacteria used for DNA probes are provided in **Table 1**.

**Table 1:** Bacterial species used to make oligonucleotide DNA probes. The mean bacterial count and mean bacterial percent were averaged over the study population of 8,173 adolescents.

				Numbers/ml x 10^−5^	Percent
Bacterial Name	Abbreviation	Phylum	ATCC	(MeanN ±S.D.)	(MeanN ±S.D.)
*Actinomyces gerencseriae*	*A*. *gerencseriae*	Actinobacteria	23860	1.95 ± 2.08	1.53 ± 0.90
*Actinomyces israelii*	*A*. *israelii*	Actinobacteria	12102	1.36 ± 1.39	1.03 ± 0.69
*Actinomyces naeslundii*^*a*^	*A*. *naeslundii*	Actinobacteria	12104	1.92 ± 1.71	1.68 ± 1.04
*Actinomyces odontolyticus (serotype I)*	*A*. *odontolyticus*	Actinobacteria	17929	4.25 ± 4.89	3.24 ± 1.91
*Actinomyces viscosus*^*b*^	*A*. *viscosus*	Actinobacteria	43146	2.10 ± 2.24	1.63 ± 1.05
*Aggregatibacter actinomycetemcomitans* (serotypes a & b)^c^	*A*. *actinomycetemcomitans*	Proteobacteria	43718 (Y4) & 29523	1.22 ± 1.09	1.06 ± 0.73
*Campylobacter gracilis*	*C*. *gracilis*	Proteobacteria	33236 (1084)	0.26 ± 0.81	0.19 ± 0.29
*Campylobacter rectus*	*C*. *rectus*	Proteobacteria	33238 (371)	0.97 ± 1.84	0.80 ± 1.38
*Campylobacter showae*	*C*. *showae*	Proteobacteria	51146	1.45 ± 1.56	1.17 ± 0.78
*Capnocytophaga gingivalis*	*C*. *gingivalis*	Bacteroidetes	33624 (27)	1.69 ± 1.85	1.34 ± 0.89
*Capnocytophaga ochracea*	*C*. *ochracea*	Bacteroidetes	(25)	1.52 ± 1.11	1.35 ± 0.68
*Capnocytophaga sputigena*	*C*. *sputigena*	Bacteroidetes	33612 (4)	2.51 ± 2.22	2.29 ± 1.72
*Eikenella corrodens*	*E*. *corrodens*	Proteobacteria	23834	12.11 ± 79.35	7.35 ± 5.73
*Fusobacterium nucleatum subsp*. *nucleatum*	*F*. *nuc*. *nuc*.	Fusobacteria	25586	1.96 ± 8.92	1.40 ± 1.36
*Fusobacterium nucleatum subsp*. *polymorphum*	*F*. *nuc*. *polymorph*.	Fusobacteria	10953	1.55 ± 1.70	1.28 ± 0.70
*Fusobacterium nucleatum subsp*. *vincentii*	*F*. *nuc*. *vinc*.	Fusobacteria	49256	1.62 ± 8.51	1.19 ± 2.18
*Fusobacterium periodonticum*	*F*. *periodonticum*	Fusobacteria	33693	2.63 ± 4.38	2.02 ± 1.49
*Gemella morbillorum*	*G*. *morbillorum*	Firmicutes	27824	1.78 ± 1.63	1.50 ± 0.83
*Lachnoanaerobaculum saburreum*^*d*^	*L*. *saburreum*	Firmicutes	33271	1.16 ± 0.84	1.03 ± 0.58
*Leptotrichia buccalis*	*L*. *buccalis*	Fusobacteria	14201	1.10 ± 2.73	1.02 ± 0.96
*Neisseria mucosa*	*N*. *mucosa*	Proteobacteria	19696	13.99 ± 14.24	12.17 ± 6.42
*Parvimonas micra*	*P*. *micra*	Firmicutes	33270	0.94 ± 0.76	0.91 ± 0.69
*Peptostreptococcaceae nodatum*^*e*^	*P*. *nodatum*	Firmicutes	33099	0.45 ± 0.47	0.37 ± 0.38
*Porphyromonas gingivalis*	*P*. *gingivalis*	Bacteroidetes	33277	2.54 ± 3.01	2.35 ± 2.92
*Prevotella intermedia*	*P*. *intermedia*	Bacteroidetes	25611	2.04 ± 5.36	1.44 ± 1.07
*Prevotella melaninogenica*	*P*. *melaninogenica*	Bacteroidetes	25845	9.15 ± 8.86	7.27 ± 4.09
*Prevotella nigrescens*	*P*. *nigrescens*	Bacteroidetes	33563	3.89 ± 4.32	2.96 ± 1.72
*Propionibacterium acnes* (serotypes I & II)	*P*. *acnes*	Actinobacteria	11827 & 11828	0.55 ± 0.60	0.41 ± 0.35
*Selenomonas noxia*	*S*. *noxia*	Firmicutes	43541	2.06 ± 1.85	1.72 ± 0.89
*Streptococcus anginosus*	*S*. *anginosus*	Firmicutes	33397	1.17 ± 1.04	0.95 ± 0.53
*Streptococcus constellatus*	*S*. *constellatus*	Firmicutes	27823 (M32b)	1.72 ± 1.78	1.47 ± 0.99
*Streptococcus gordonii*	*S*. *gordonii*	Firmicutes	10558	2.15 ± 2.10	1.70 ± 0.82
*Streptococcus intermedius*	*S*. *intermedius*	Firmicutes	27335	1.76 ± 1.62	1.47 ± 0.88
*Streptococcus mitis*	*S*. *mitis*	Firmicutes	49456	11.53 ± 11.42	10.0 ± 5.77
*Streptococcus mutans*	*S*. *mutans*	Firmicutes	25175	1.44 ± 1.57	1.26 ± 0.79
*Streptococcus oralis*	*S*. *oralis*	Firmicutes	35037	6.85 ± 7.14	5.58 ± 2.57
*Streptococcus salivarius*	*S*. *salivarius*	Firmicutes	27945	5.01 ± 7.56	3.72 ± 2.46
*Streptococcus sanguinis*	*S*. *sanguinis*	Firmicutes	10556	3.00 ± 2.68	2.55 ± 1.34
*Tannerella forsythia*	*T*. *forsythia*	Bacteroidetes	43037 (338)	0.61 ± 0.55	0.48 ± 0.35
*Treponema denticola*	*T*. *denticola*	Spirochaetes	(B1)	0.61 ± 0.67	0.51 ± 0.52
*Treponema socranskii*	*T*. *socranskii*	Spirochaetes	(D40DR2) (S1)	0.66 ± 0.95	0.57 ± 0.91
*Veillonella parvula*	*V*. *parvula*	Firmicutes	10790	7.31 ± 7.43	6.04 ± 3.04

^a^ Formerly *Actinomyces naeslundii 1*.

^b^ Formerly *Actinomyces naeslundii 2*.

^c^ Formerly *Actinobacillus actinomycetemcomitans*.

^d^ Formerly *Eubacterium saburreum*.

^e^ Formerly *Eubacterium nodatum*.

### Salivary glucose analysis

Methods for measuring salivary glucose concentration have been previously described [24]. Briefly, saliva samples were weighed and then centrifuged to remove particulate debris, and a 30-μL aliquot of saliva supernatant was assayed. The assay used the glucose oxidase method with a fluorescent dye (Glucose Colorimetric/Fluorometric Assay Kit #K606-100, BioVision, Inc, Mountain View, California, USA) measured at Ex/Em -535/590 nm and adapted to work on a Tecan Freedom EVO^®^ 150 robotic processor with an 8-channel liquid handling arm (Tecan Group Ltd, Männedorf, Switzerland). Fluorescence was measured by a spectrophotometer (Infinite^®^ 200 Pro, Tecan Group Ltd, Männedorf, Switzerland) using reverse 96-well plate reading mode. The 3 sigma detection limit of the glucose assay was 0.002 mg/dL. Standards of 0.12, 0.24, 0.48 and 0.96 mg/dL were assayed in triplicate on each run.

### Statistical and analytical

Dental caries were evaluated as a percentage by counting the number of teeth (primary and permanent) with visible caries, or cavities plus fillings, and dividing by the total number of teeth. Gingivitis was evaluated as a percentage by counting the number of red sites around both deciduous and permanent teeth and dividing by the total number of sites in the mouth (four total sites/tooth). Differences in discrete measures were tested by chi-square analysis. Differences in continuous clinical variables (age, percent carious teeth, percent gingival redness, BMI, average sleep duration, and fitness level) were tested by two-sample t-test. Differences between parameters related to salivary bacterial composition were tested using the Kruskal-Wallis method.

**Fig 1:**
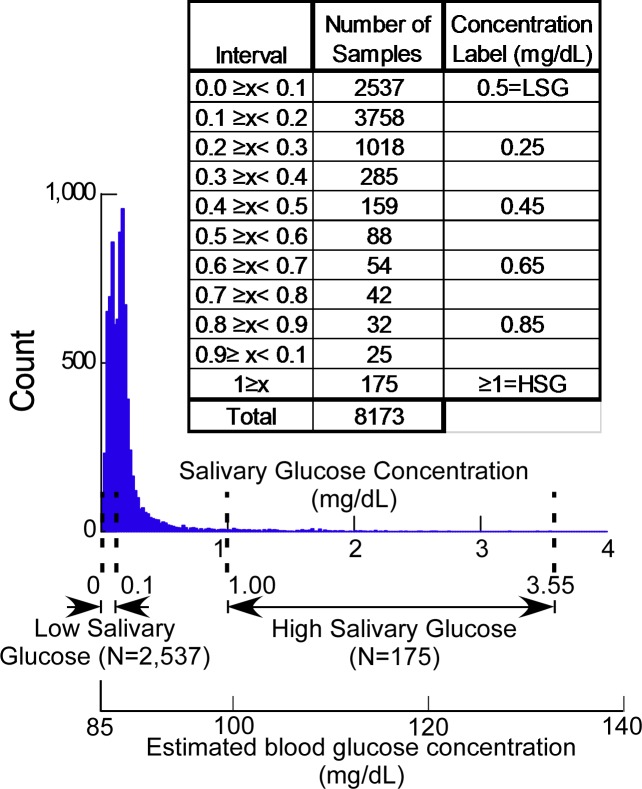
Distribution of salivary glucose levels measured in 10-year old Kuwaiti adolescents. The fasting salivary glucose concentration of 97.8% of samples assayed was below 1 mg/dL (normal healthy range). Fasting salivary glucose concentrations above 1 mg/dL correlate with plasma glucose concentrations ≥100 mg/dL. The inset table defines intervals and number of samples used for median value computation (x = salivary glucose concentration). High salivary glucose (HSG) intervals, low salivary glucose (LSG) intervals, and estimated blood concentration (for plasma concentrations ≥ 84.8 mg/dL, Plasma = 13.5*Saliva+84.8 [24]) are illustrated.

The total number of bacteria in each sample was computed as the sum of the bacterial count for each of the 42 bacterial species probes used. Percentages of bacteria were calculated for each of the 42 species by dividing the bacterial count for each species by the sum of all bacteria counts measured in each sample. Bacterial numbers and percentages were computed as median values for 11 salivary glucose intervals which uniformly cover the range of values measured with LSG and HSG defined at the left and right extremes. These intervals (***Fig* 1**) were 0≥x< 0.1, 0.1≥x<0.2… 0.9≥x<1.0 and ≥1.0.

We conducted subgroup analyses comparing results from the group (n = 2,537, 31%) of participants with low salivary glucose (LSG), which was defined as a salivary glucose concentration of less than 0.1 mg/dL, with those of the group of participants (n = 175, 2.1%) with high salivary glucose (HSG), which was defined as a salivary glucose concentration greater than or equal to 1.0 mg/dL. Differences in the total bacterial count per mL in samples between participants with LSG and those with HSG were tested for their ability to predict obesity by random forest analyses (Salford Systems, San Diego, CA, USA) that included dental caries, gingivitis, and BMI in the model, as well as by univariate receiver operating characteristic (ROC) analysis using linear support vector machines algorithms for multivariate analysis [30]. Statistically significant values were accepted when p< 0.001, the Bonferroni adjusted p-value to obtain a familywise error rate of p<0.05 with 42 comparisons.

## Results

### Patient characteristics

Saliva samples and clinical data were collected from 8,173 Kuwaiti adolescents aged 10.0 ± 0.67 years (**Table 2**). The mean saliva collection time for all participants was 8.1 ± 0.7 minutes. There were no significant age or BMI differences between sexes. The total study population contained significantly more girls (61.1%) than boys (38.9%), and a significantly higher percentage of boys were obese (38.2%) than were girls (31.0%). The average salivary flow rate was also significantly higher in boys (28.22 ± 17.06 mL/h) than girls (24.64 ± 14.36 mL/h). The mean salivary glucose concentration was significantly higher among boys (0.22 ± 0.28 mg/dL) than girls (0.18 ± 0.22 mg/dL). Boys had a larger percentage of carious or filled teeth and more untreated carious teeth than girls. Gingival redness was high in both boys and girls but was slightly higher among the girls.

**Table 2:** Demographic and clinical variables for the total study population of 8,173 adolescents by sex.

Variable	Overall	Male	Female	*P*-value
N (%)	8,173 (100%)	3,181 (38.9%)	5,068 (61.1%)	<0.001
Obese (%)	2,789 (34.1%)	1,215 (38.2%)	1,574 (31.0%)	<0.001
Age (y)	10.00 ± 0.67	9.99 ± 0.67	10.00 ± 0.67	0.5
Salivary flow rate (mL/h)	26.02 ± 15.56	28.22 ± 17.06	24.64 ± 14.36	<0.001
Salivary glucose (mg/dL)	0.19 ± 0.24	0.22 ± 0.28	0.18 ± 0.22	<0.001
BMI (kg/m^2^)	20.89 ± 5.21	20.92 ± 5.37	20.83 ± 5.12	0.5
Carious or filled teeth (%)	10.92 ± 10.41	11.87 ± 10.82	10.43 ± 10.16	<0.001
Carious teeth (%)	6.97 ± 8.96	7.73 ± 9.35	6.58 ± 8.73	<0.001
Red gingival sites (%)	74.85 ± 21.16	73.47 ± 21.78	75.27 ± 21.07	<0.001
Total number of bacteria (x 10^5^/mL)	124 ± 126	122 ± 166	126 ± 95	0.2

### Distribution of salivary glucose concentrations

Salivary glucose concentrations in study subjects were distributed as illustrated in ***Fig* 1**. HSG (salivary glucose concentration >0.1 mg/dL) was found in 2.1% of the population (n = 175). LSG (salivary glucose concentration ≤0.1 mg/dL) was found in 31% of the population (n = 2,537).

### Salivary glucose concentration differences and clinical characteristics

We compared clinical characteristics among the 2,712 participants who exhibited either LSG or HSG (**Table 3**). The analysis revealed striking differences in oral disease parameters between the LSG and HSG groups, but only relatively small differences in parameters related to metabolic disease. Among the oral tissue parameters, both dental caries and gingivitis were significantly increased with HSG. However, measures of obesity (BMI and waist circumference), fitness level, weekday sleep duration, and blood pressure were not significantly different between the groups. Among those adolescents in the HSG group, 57 (33%) were obese, 52 (30%) had high blood pressure, and 29 (17%) both had high blood pressure and were obese (**Table 4**). Of note, 95 adolescents with HSG (54%) were of normal weight and did not have high blood pressure, demonstrating that HSG was often present in the saliva of adolescents who did not exhibit clinical signs of metabolic disease. In the LSG group, 868 (34%) were obese, 589 (23%) had high blood pressure, and 346 (14%) both had high blood pressure and were obese.

**Table 3:** Clinical characteristics (mean ± standard deviation) of adolescents with LSG and HSG.

Clinical characteristics	LSG (n = 2537)	HSG (n = 175)	% difference	Hypothesis testing (p, t)
Carious teeth (% decayed)	5.43 ± 7.71	8.35 ± 8.55	53.9	<0.001, -4.40
Gingival redness (% red)	72.55 ± 19.94	78.58 ± 19.45	8.3	<0.001, -3.96
Total number of bacteria (x 10^−5^/ml)	123.97 ± 86.64	67.61 ± 52.39	-45.5	<0.001, 13.05
Saliva flow rate (ml/h)	25.83 ± 15.61	27.93 ± 16.79	8.2	0.1, -1.61
BMI (kg/m^2^)	21.12 ± 5.22	20.65 ± 5.45	-2.2	0.3, 1.10
Waist circumference ((cm)	68.09 ± 21.56	67.06 ± 12.41	-1.5	0.3, 1.00
Fitness (beats/min)	25.29 ± 19.17	25.61 ± 22.01	1.3	0.9, -0.19
Sleep (hr)	8.82 ± 1.60	9.09 ± 1.67	3	0.04, -2.06
Diastolic blood pressure (mmHg)	73.94 ± 13.25	75.00 ± 14.10	1.4	0.3, -0.97
Systolic blood pressure (mmHg)	109.10 ± 16.61	109.73 ± 18.36	0.6	0.7, -0.44
Age (y)	10.16 ± 0.66	9.89 ± 0.70	-2.6	<0.001, 4.89

**Table 4:** Number of adolescents with HSG or LSG and high blood pressure and or obesity.

Body weight & salivary glucose category	Normal BP	High BP	Total
Normal weight-HSG	95 (54%)	23 (13%)	118 (67%)
Obese-HSG	28 (16%)	29 (17%)	57 (33%)
Total-HSG	123 (70%)	52 (30%)	175 (100%)
Normal weight-LSG	1426 (56%)	243 (10%)	1669 (66%)
Obese-LSG	522 (21%)	346 (14%)	868 (34%)
Total-LSG	1948 (77%)	589 (23%)	2537 (100%)

Bacterial species counts in saliva samples were measured by hybridization with whole genomic probes (**Table 1)**. Over 50% of the assayed bacteria were accounted for by seven species (*N*. *mucosa*, *E*. *corrodens*, *S*. *mitis*, *P*. *melaninogenica*, *V*. *parvula*, *S*. *oralis* and *S*. *salivarius*). Bacterial species present with the highest counts in the saliva samples included *N*. *mucosa*, *E*. *corrodens* and *S*. *mitis*, each present at mean concentrations of greater than 10^6^/mL. Bacteria with the lowest counts included *P*. *nodatum* and *C*. *gracilis*, each present at mean concentrations of less than 0.5 x 10^5^/mL.

### Salivary bacterial load and species counts are altered with increasing salivary glucose concentration

**Fig 2:**
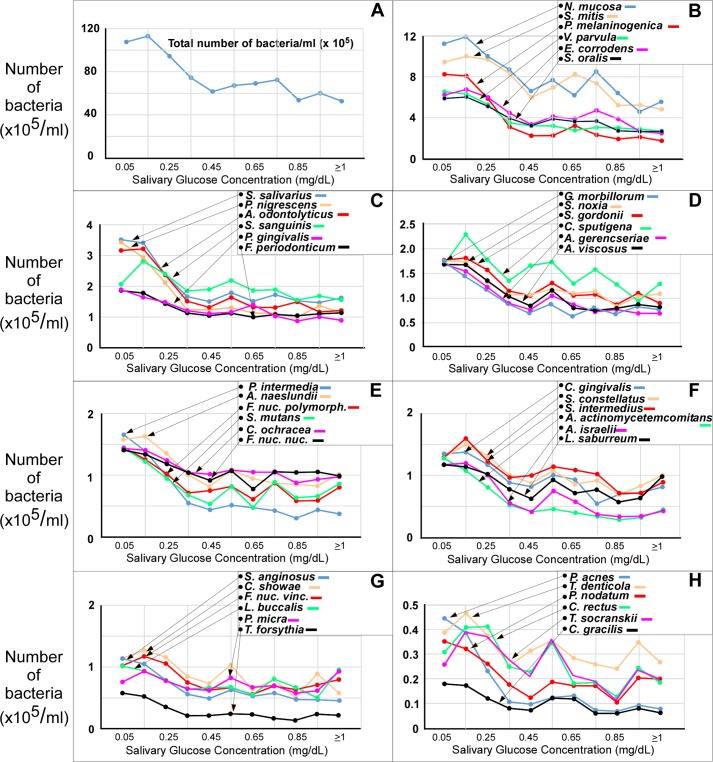
Bacterial count (median number x 10^5^/mL) in saliva with increasing salivary glucose concentration. The total bacterial load (sum of the 42 species measured) is shown in (A). The mean bacterial count of the 42 bacterial species evaluated are shown in (B) through (H).

Both the total bacterial load and the bacterial count of almost every species tested in this study decreased with increasing salivary glucose concentration (***Fig* 2**). The total bacterial load (A) was 107.6 x 10^5^/mL for the salivary glucose concentration interval 0≥x< 0.1 (displayed at 0.05), rose slightly to 113.1 x 10^5^/mL for the interval 0.1≥x< 0.2, and then fell to 52.7 x 10^5^/mL for the interval x≥1. A similar profile is seen for the predominate species *N*. *mucosae* (B). Other species (B) through (H) decreased in count, with differing sensitivity to increasing salivary glucose concentration. The only bacterial species to increase in count (p>0.001, NS) with increasing salivary glucose concentration was *P*. *micra* (***Fig* 2G**).

The median bacterial count under HSG and LSG conditions for each species are listed in **Table 5**. About 88% of the species (n = 37) were found to have statistically significant (*p*≤0.001) changes in count between the LSG and HSG conditions. The order of reduction was in the direction of the reported aciduric strength of the bacterial species, with *Prevotella spp*. being most sensitive and *S*. *mutans* among the most resistant.

**Table 5:** Median bacterial counts (number/mL x 10^−5^) in LSG and HSG conditions, sorted by univariate area under the curve (AUC). Percent difference was computed as [100 x (HSG-LSG)/LSG] between the HSG group and the LSG group. Negative values represent a reduction in bacterial count. The random forest ROC area under the curve was 0.935. Source data is listed in S1 Table.

			Bacterial numbers (median x 10^5^/ml)		
Bacteria	Random Forest Importance	Univariate AUC	Low salivary glucose (LSG)	High salivary glucose (HSG)	% difference
*P*. melaninogenica**	81.3	0.83	8.25	1.78	-78.5
*P*. nigrescens**	79.5	0.81	3.41	1.12	-67.1
*P*. intermedia**	90.7	0.81	1.66	0.37	-77.5
*A*. actinomycetemcomitans**	100.0	0.79	1.29	0.45	-64.6
*P*. acnes**	34.9	0.78	0.44	0.08	-82.5
*A*. odontolyticus**	24.6	0.77	3.15	1.19	-62.2
*A*. gerencseriae**	38.3	0.76	1.68	0.68	-59.5
*G*. morbillorum**	43.1	0.75	1.77	0.77	-56.2
*S*. salivarius**	12.7	0.75	3.51	1.62	-53.8
*A*. viscosus**	29.1	0.74	1.67	0.81	-51.7
*E*. corrodens**	11.8	0.74	6.30	2.57	-59.2
*A*. israelii**	31.0	0.74	1.18	0.42	-64.0
*V*. parvula**	16.4	0.73	6.56	2.73	-58.4
*N*. *mucosa*	12.8	0.73	11.21	5.57	-50.3
*F*. *periodonticum*	15.2	0.73	1.86	1.14	-38.9
*S*. mutans**	22.4	0.73	1.43	0.85	-40.5
*C*. gingivalis**	5.4	0.72	1.35	0.81	-39.9
*S*. oralis**	17.2	0.72	5.84	2.63	-55.0
*A*. naeslundii**	23.4	0.71	1.58	1.01	-35.9
*S*. anginosus**	22.9	0.71	1.14	0.46	-59.8
*F*. *nuc*. *polymorph*.**	27.0	0.71	1.44	0.80	-44.7
*S*. mitis**	10.5	0.71	9.38	4.78	-49.0
*T*. forsythia**	6.4	0.70	0.57	0.21	-63.1
*S*. noxia**	9.7	0.70	1.76	1.09	-38.2
*P*. gingivalis**	59.7	0.69	1.88	0.88	-53.1
*C*. ochracea**	21.4	0.69	1.42	0.96	-32.4
*C*. showae**	14.0	0.69	1.13	0.57	-49.9
*S*. gordonii**	13.3	0.69	1.76	0.89	-49.5
*F*. *nuc*. *nuc*.**	26.1	0.68	1.40	0.98	-29.8
*C*. gracilis**	8.6	0.68	0.18	0.06	-66.5
*P*. nodatum**	9.6	0.64	0.35	0.20	-43.0
*L*. saburreum**	16.1	0.62	1.18	0.99	-16.3
*S*. constellatus**	24.7	0.61	1.31	1.02	-22.2
*S*. intermedius**	27.4	0.61	1.29	0.89	-30.9
*S*. sanguinis**	11.5	0.59	2.07	1.56	-24.5
*F*. *nuc*. *vinc*.**	26.1	0.59	1.01	0.79	-22.2
*C*. rectus**	30.9	0.58	0.31	0.19	-39.6
T. denticola*	9.6	0.57	0.39	0.27	-30.7
C. sputigena*	23.4	0.56	1.68	1.28	-23.9
P. micra*	51.3	0.55	0.75	0.92	22.8
*T*. *socranskii*	35.3	0.54	0.25	0.20	-21.2
*L*. *buccalis*	22.3	0.52	1.01	0.95	-6.0
**Clinical measures**					
Age (y)**	13.2	0.62	10.1	9.7	-4.0
% of teeth with untreated decay**	0.8	0.61	3.8	7.1	86.8
% of red gingival sites**	0.0	0.59	74.2	83.3	12.3
Weekday sleep*	0.0	0.55	9	9	0.0
BMI	0.0	0.54	19.9	18.9	-5.0
Fitness*	0.0	0.50	24.5	24	-2.0

*p<0.05

**p<0.001

### Salivary bacterial load predicts salivary glucose concentration

Using a random forest analysis and a multivariate ROC analysis, the overall bacterial load was found to accurately predict HSG (**Table 5)**. The area under the curve was 0.949 by random forest analysis and 0.935 by multivariate ROC analysis. When clinical measures (sex, BMI, fitness level, sleep duration, percentage of carious teeth, and gingival redness) were included, they were given a random forest importance of less than or equal to 13.2 in prediction of HSG. In the random forest analysis, the most predictive individual bacterial species was *A*. *actinomycetemcomitans*, for which a decrease in bacterial count predicted HSG (AUC = [0.79]). In the multivariate ROC analysis, the most predictive individual bacterial species was *P*. *melaninogenica*, for which a decrease in bacterial count predicted HSG (AUC = 0.83).

### Relative bacterial species frequency is altered with high salivary glucose

We next determined if the relative frequency of each bacterial species to the overall mean salivary bacterial count differed between HSG and LSG conditions. For this analysis, we considered the bacterial count for each species under conditions of LSG as representative of the normal, healthy state. We then computed the percent difference in bacterial count for each species between the LSG and HSG conditions (**Table 6)**. A significant change in percentage was seen for 26 (62%) of the bacterial species under conditions of HSG. Of these,15 (36%) species decreased in percentage, while 11 (27%) bacterial species increased in percentage. The remaining 16 (38%) did not significantly change in percentage between the LSG and HSG conditions. The *Prevotella spp* exhibited the largest percentage reduction in the HSG condition, while the percentage of *S*. *mutans* did not significantly change between conditions. *P*. *micra* exhibited both the highest univariate AUC (0.82) and was also the most important species in the random forest analysis in prediction of HSG.

**Table 6:** Median salivary bacterial percentages under conditions of LSG and HSG sorted by univariate AUC. Difference was computed as [(HSG-LSG)], such that negative values represent a reduction in percentage as salivary glucose increases. The random forest ROC area under the curve = 0.968. Source data is listed in S2 Table.

			Median Bacteria Percentage		
**Bacteria % decrease with high glucose**	**Random Forest Importance**	**Univariate AUC**	**Low Salivary Glucose (LSG)**	**High Salivary Glucose (HSG)**	**Difference**
*P*. melaninogenica**	53.8	0.80	7.51	3.71	-3.80
*P*. nigrescens**	78.0	0.76	3.22	1.91	-1.31
*P*. intermedia**	68.5	0.76	1.55	0.75	-0.80
*P*. acnes**	24.7	0.74	0.41	0.16	-0.25
*A*. actinomycetemcomitans**	52.0	0.68	1.19	0.79	-0.40
*A*. gerencseriae**	74.3	0.67	1.58	1.20	-0.39
*A*. israelii**	16.6	0.65	1.07	0.74	-0.32
*A*. odontolyticus**	6.6	0.64	2.99	2.44	-0.55
*A*. viscosus**	14.6	0.61	1.61	1.30	-0.31
*T*. forsythia**	8.6	0.60	0.49	0.32	-0.17
*S*. salivarius**	8.5	0.60	3.40	3.01	-0.39
*G*. morbillorum**	18.3	0.60	1.63	1.41	-0.22
*C*. gracilis**	4.5	0.58	0.16	0.12	-0.04
*S*. anginosus**	16.9	0.58	0.99	0.85	-0.14
*E*. corrodens**	6.5	0.57	6.14	5.30	-0.84
*P*. gingivalis**	31.5	0.57	1.63	1.19	-0.44
*C*. gingivalis*	2.9	0.56	1.28	1.13	-0.15
*C*. showae*	10.1	0.55	1.02	0.91	-0.11
*F*. periodonticum*	6.3	0.55	1.88	1.78	-0.11
*V*. *parvula*	8.5	0.54	6.00	5.56	-0.43
*S*. *oralis*	8.0	0.53	5.37	5.33	-0.04
*F*. *nuc*. *polymorph*.	10.6	0.51	1.39	1.37	-0.01
*C*. *rectus*	29.7	0.51	0.28	0.27	-0.01
*N*. *mucosa*	9.0	0.50	11.30	10.83	-0.47
**Bacteria % increase with high glucose**					
*P*. micra**	100.0	0.82	0.73	1.51	0.78
*L*. buccalis**	39.0	0.74	0.91	1.54	0.63
*C*. sputigena**	58.9	0.73	1.63	2.52	0.89
*S*. sanguinis**	37.9	0.71	1.96	3.05	1.09
*S*. constellatus**	55.8	0.64	1.20	1.64	0.44
*F*. *nuc*. *vinc*.**	29.8	0.63	0.86	1.15	0.28
*S*. intermedius**	41.4	0.61	1.20	1.48	0.28
*L*. saburreum**	8.2	0.60	1.04	1.20	0.15
*T*. denticola**	17.9	0.59	0.36	0.52	0.16
*T*. socranskii**	30.1	0.59	0.23	0.34	0.11
*C*. ochracea**	6.6	0.58	1.34	1.55	0.21
*F*. *nuc*. *nuc*.*	7.8	0.57	1.31	1.42	0.10
*S*. noxia*	14.7	0.56	1.67	1.89	0.22
*S*. *mitis*	7.7	0.53	8.61	9.27	0.66
*S*. *gordonii*	5.9	0.52	1.63	1.72	0.10
*P*. *nodatum*	5.6	0.51	0.33	0.37	0.04
*S*. *mutans*	18.7	0.51	1.33	1.40	0.07
*A*. *naeslundii*	3.5	0.51	1.49	1.61	0.12
**Clinical measures**					
Age (y)**	13.4	0.62	10.1	9.7	-4.0
% of teeth with untreated decay**	1.2	0.61	3.8	7.1	86.8
% of red gingival sites**	0.1	0.59	74.2	83.3	12.3
Weekday sleep*	0.7	0.55	9	9	0.0
BMI	2.2	0.54	19.9	18.9	-5.0
Fitness	0.0	0.50	24.5	24	-2.0

*p<0.05

**p<0.001

### Phylum counts and relative frequency are altered with high salivary glucose

We also analyzed phylum counts and found that all decreased under HSG conditions when compared with counts seen under LSG conditions (**Table 7**). The phylum *Bacteroidetes* decreased to the greatest extent. Relative phyla frequencies under conditions of LSG and HSG are listed in **Table 8**. The phylum *Bacteroidetes* decreased to the greatest extent (-5.7%) under HSG conditions, whereas the phylum *Firmicutes* increased by the greatest extent (4.3%) under HSG conditions.

**Table 7:** Median phyla counts under conditions of LSG and HSG. Data are sorted by % difference [100 x (HSG-LSG)/LSG] such that negative values represent a reduction under HSG conditions. The random forest ROC area under the curve for phyla = 0.868. Source data is listed in S1 Table.

			Bacteria Number (Median x 10^5^/mL)			
Phylum	Random forest Importance	Univariate AUC	Low salivary glucose (LSG)	High salivary glucose (HSG)	% Difference	Hypothesis testing (p,χ^2^)
*Bacteroidetes*	66.6	0.79	21.8	8.6	-60.8	<0.0001, 170
*Actinobacteria*	100.0	0.77	10.3	4.2	-59.0	<0.0001, 135
*Proteobacteria*	20.0	0.71	20.8	10.4	-50.1	<0.0001, 104
*Firmicutes*	37.0	0.69	42.5	22.6	-46.8	<0.0001, 89
*Fusobacteria*	20.7	0.66	6.9	4.9	-28.6	<0.0001, 52
*Spirochaetes*	49.0	0.53	0.7	0.6	-7.7	0.3, 1.3

**Table 8:** Median phyla frequencies under conditions of LSG and HSG. Data are sorted by difference (HSG-LSG), such that negative values represent a decrease in percentage under HSG conditions. The random forest ROC area under the curve for phyla = 0.884. Source data is listed in S2 Table.

			Median Bacteria %			
Phyla % decreasing with high glucose	Random forest Importance	Univariate AUC	Low salivary glucose (LSG)	High salivary glucose (HSG)	Difference	Hypothesis testing (p,χ^2^)
*Bacteroidetes*	100.0	0.77	20.3	14.6	-5.7	<0.001, 117
*Actinobacteria*	45.5	0.69	9.6	7.6	-2.0	<0.001, 60
*Proteobacteria*	21.4	0.51	20.7	20.4	-0.3	0.3, 0.6
Phyla % increasing with high glucose						
*Firmicutes*	36.2	0.64	39.9	44.2	4.3	<0.001, 23
*Spirochaetes*	70.8	0.62	0.6	1.0	0.4	<0.001, 37
*Fusobacteria*	45.1	0.60	6.7	8.1	1.4	<0.001, 32

## Discussion

In the current study of samples of whole saliva from 8,173 Kuwaiti adolescents, we conducted a checkerboard assay to analyze the salivary microbiota, comparing differences in overall bacterial load and the count and relative frequency of 42 different bacterial species between the group of samples with LSG and the group with HSG. We found that over 50% of the assayed bacteria overall were accounted for by seven species (*N*. *mucosa*, *E*. *corrodens*, *S*. *mitis*, *P*. *melaninogenica*, *V*. *parvula*, *S*. *oralis*, *and S*. *salivarius*). The overall mean salivary bacterial count decreased with increasing salivary glucose concentration. Indeed, overall salivary counts more accurately predicted HSG in this cohort than did clinical measures, including sex, BMI, fitness level, sleep duration, percentage of decayed teeth, and gingival redness. When considering the individual bacterial species, 88% of the 42 species exhibited a statistically significant difference in count between LSG and HSG conditions, and 62% exhibited a statistically difference in relative frequency between LSG and HSG conditions. For those species that displayed a reduced bacterial count and/or frequency under HSG conditions, the magnitude of the difference reflected the growth sensitivity of each bacterial species to an acidic environment.

**Fig 3:**
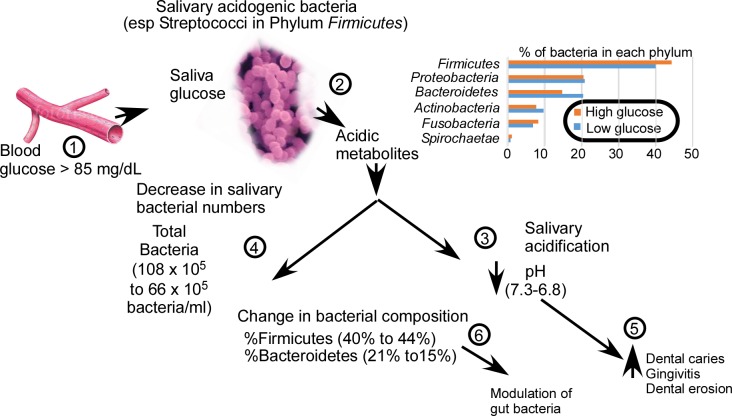
Proposed hypothesis for salivary microbial changes in response to high salivary glucose concentration. The central theme of this hypothesis is that hyperglycemia changes the oral microbial environment by salivary acidification. Numbers refer to points made in discussion. The bar graph represents data from Table 8.

Since glucose is a well-known energy source for many oral bacteria, it is no surprise that alterations in the salivary glucose concentration would affect the salivary microbiome. However, we did not expect overall bacterial counts to decrease with increasing concentrations of glucose. This is particularly true given that many of the species most affected by increases in salivary glucose concentration, including *P*. *nigrescens*, *P*. *intermedia*, and *P*. *melaninogenica*, are asaccharolytic. Therefore, we propose the following hypothesis to account for the observed changes (***Fig* 3**).

The central theme of our hypothesis is that hyperglycemia changes the oral microbial environment by salivary acidification. The first step in this process (***Fig* 3, Point 1**) is the increase in salivary glucose concentration that occurs with hyperglycemia in blood. Glucose transporters have been found in both the acinar and ductal cells of rodent salivary glands [31]. The positioning of the GLUT1, GLUT4, and SGLT1 transporters suggests that glucose may be transported from blood to saliva into the collecting duct to sustain ductal cellular metabolism. Meta-analyses of controlled studies demonstrate elevated salivary glucose concentrations in patients with both type 1 and type 2 diabetes [22]. We have previously shown that the salivary glucose concentration can be related to the blood glucose concentration via a threshold model [24]. When the blood glucose concentration exceeds 84.8 mg/dL, glucose begins to appear in saliva. When the blood glucose concentration reaches hyperglycemic levels (≥100 mg/dL), the salivary concentration becomes ≥1 mg/dL. This threshold effect is hypothesized to occur when glucose transport exceeds ductal cell uptake in conditions of hyperglycemia, such as in T2D. When glucose appears at a high concentration in saliva, one would expect an increased level of bacterial synthesis of acidic metabolites (***Fig 3*, Point 2**), given that between 39.9% and 44.2% of salivary bacteria are acidogenic *Firmicutes* (**Table 8**). Indeed, the presence of dietary carbohydrates is well recognized as a stimulus for lowered salivary pH due to incomplete carbohydrate metabolism by oral acidogenic bacteria [32]. Further, the rapid lowering of pH in dental biofilms following exposure to glucose rinses (the “Stephan curve”) has been known for over 70 years [33]. Many investigators have reported that the salivary pH is lower in adults and adolescents with diabetes than it is in healthy adults and adolescents [34–36]. Reported differences are of sufficient magnitude to be measured by pH paper [37, 38], and to have been proposed as a simple screening measure for detection of metabolic syndrome or diabetes [39]. Therefore, according to our model, blood hyperglycemia in patients with T2D will lead to elevated levels of salivary glucose concentration, an increased synthesis of acidic metabolites by oral acidogenic bacteria, and salivary acidification (***Fig 3*, Point 3**).

Acidification is proposed to interfere with bacterial reproduction (***Fig* 3, Point 4**), altering the relative bacterial species frequency and count of the oral microbiome. This proposal is consistent with the reduction of oral bacterial growth under conditions of lowered pH that has been reported in many studies [40–43]. Studies of acid resistance in oral bacteria [40, 41] indicate that *S*. *mutans* is more acid resistant than *S*. *salivarius*, and *A*. *viscosus*, which is also seen in **Table 5**. About 35% to 75% of *S*. *oralis* and *Actinomycetes* populations are reported to be killed within 1 hour at pH 4.2–4.4 [42]. Other studies comparing oral bacterial growth on blood agar versus acid agar at pH 5 [43] found that the phyla *Bacteroidetes*, *Fusobacteria*, and *Proteobacteria* were not significantly enhanced on pH 5 agar. Our study indicates that, of all the phyla tested, *Bacteroidetes* is most sensitive to increased salivary glucose concentration, though the species isolated were non-saccharolytic.

It is known that salivary acidification is a major factor in the development of both dental caries [44] and gingivitis [45, 46] because it alters the oral microbiome to favor caries-associated bacterial species, such as *Bifidobacterium dentium*, *Bifidobacterium longum*, and *S*. *mutans* [40]. An elevated salivary glucose concentration also appears, in our study, to increase the risk of dental erosion, dental caries, and gingivitis (***Fig 3*, Point 5**). We observed that adolescents with HSG had almost twice the percentage of carious teeth than did adolescents with LSG (**Table 3**). We also observed that adolescents with HSG had a significantly increased percentage of gingival redness compared with adolescents with LSG. This is in line with data from a study in which increased dental erosion was reported in patients with hyperglycemia and renal disease [47]. Consumption of sugar has long been associated with dental decay, even though consumed sugars are rapidly cleared from the oral cavity, with a halftime of about 2.2 minutes [32]. We propose that it is underlying hyperglycemia that is the real cause of dental decay, because patients with hyperglycemia can have persistent salivary glucose concentrations exceeding 1 mg/dL.

Both gingivitis and dental caries have been related to the development of obesity [32, 48] and T2D [49, 50]. Those investigating the role of bacteria in the gastrointestinal tract as a cause of obesity (“infectobesity”) have generally sought identification of specific pathogenic bacteria. These observations, though somewhat variable, have reported that obesity is associated with high proportions of *Firmicutes* and reduced proportions of *Bacteroidetes* [51] in the oral microbiome. This is similar to what we have found (**Table 8**). Identification of specific pathogenic bacteria, however, has eluded scientific enquiry. Our study suggests that differences in gut bacterial composition between people who are obese and those of normal weight may be largely the consequence of hyperglycemia, rather than the cause (***Fig 3*, Point 6**). Also, since all gastric bacteria are introduced through the oral cavity, it is possible that a lowered salivary pH due to hyperglycemia may act as a filter to inhibit replenishment of gastric *Bacteroidetes*, while more facilely transmitting gastric *Firmicutes* species.

## Conclusions

There are reports of major changes in the bacterial frequency and/or bacterial species counts in saliva from obese individuals compared with samples from those of healthy weight. T2D has been associated with changes in bacterial diversity and frequency in supragingival plaque, but an association between T2D and changes in salivary bacterial parameters has been less clear. In considering the role of oral bacteria in obesity and the development of T2D, one is hard pressed to determine whether the observed changes to the oral microbiome are the cause of these conditions, or one of the effects. In the current study, we have confirmed that the overall salivary bacterial load, as well as the bacterial counts and relative frequencies of various bacterial species, are significantly altered in adolescents with HSG. Our current study supports the idea that increased concentrations of salivary glucose may be the root cause of these perturbations in the oral microbiome, as has been suggested by others as well [52]. One cannot rule out the possibility that a bacterial species, such as that of *P*. *micra*, or a bacterial species we did not measure by the DNA probe method, could have a direct effect on the development of obesity and T2D. However, our data support a model in which hyperglycemia associated with obesity and T2D results in an increased salivary glucose concentration, which appears to reduce the salivary pH. This, in turn, reduces the overall bacterial count of the oral microbiome and alters the relative bacterial frequencies to favor aciduric bacterial species. This sequence of events suggests a basis for the observation that hyperglycemia is associated with an increased risk of dental erosion, dental caries, and gingivitis. Further studies will elucidate the utility of analyzing salivary bacterial load and species frequency as predictors for hyperglycemia and/or T2D.

## Supporting information

S1 Table The number of bacteria in each sample (x 10^5^). (XLSX)

S2 Table The percentage of bacteria in each sample. (XLSX)

## Abbreviations

ATCC: American Type Culture Collection
BMI: Body Mass Index
HSG: high salivary glucose
LSG: low salivary glucose
T2D: type 2 diabetes
